# Effectiveness of motivational interviewing on anxiety, depression, sleep quality and quality of life in heart failure patients: secondary analysis of the MOTIVATE-HF randomized controlled trial

**DOI:** 10.1007/s11136-021-02788-3

**Published:** 2021-02-22

**Authors:** Paola Rebora, Valentina Spedale, Giuseppe Occhino, Michela Luciani, Rosaria Alvaro, Ercole Vellone, Barbara Riegel, Davide Ausili

**Affiliations:** 1 Department of Medicine and Surgery, University of Milano - Bicocca, Via Cadore 48, 20900 Monza, Italy; 2 Department of Biomedicine and Prevention, University of Rome Tor Vergata, Via Montpellier, 1, 00133 Rome, Italy; 3 Biostatistics and Bioimaging Centre, University of Milano - Bicocca, Via Cadore 48, 20900 Monza, Italy; 4 University of Pennsylvania School of Nursing, 418 Curie Boulevard, Philadelphia, PA 19104-4217 USA

**Keywords:** Heart failure, Motivational interviewing, Quality of life, Anxiety, Depression, Sleep quality

## Abstract

**Purpose:**

Anxiety, depression, poor sleep quality and lower quality of life (QOL) are associated with worse outcomes in heart failure (HF) patients. Motivational interview (MI) has been effective in different patient populations to promote self-care. However, its effect on anxiety, depression, sleep quality and QOL in HF patients is unknown. The aim of this study was to evaluate the effect of MI on anxiety, depression, sleep quality and QOL over 12 months from the intervention.

**Methods:**

This was a planned, secondary outcome analysis of the MOTIVATE-HF study, a three-arm randomized controlled trial (1:1:1) evaluating the effect of MI in improving self-care in HF patients. In Arm 1, the patient received MI, while in Arm 2, the patient and the caregiver received MI. Arm 3, the control group, received standard treatment. Endpoints were evaluated with the Hospital Anxiety and Depression Scale (HADS), the Pittsburgh Sleep Quality Index (PSQI), the 12-Item Short-Form Health Survey (SF-12) and the Kansas City Cardiomyopathy Questionnaire (KCCQ) every three months for one year.

**Results:**

We enrolled and randomized 510 HF patient and caregiver dyads (155 dyads in Arm 1, 177 dyads in Arm 2, and 178 dyads in Arm 3). A total of 238 HF patients and 235 caregivers completed the 12-month trial. No significant changes were seen in anxiety, depression and sleep quality over time among the three study arms, but disease-specific QOL improved in the intervention groups, especially in Arm 2.

**Conclusion:**

Clinicians may want to include both patients and caregivers when providing MI interventions. Further research is needed to investigate the required intensity of MI to be effective on sleep quality, anxiety and depression (ClinicalTrials.gov Identifier: NCT02894502).

**Supplementary Information:**

The online version contains supplementary material available at 10.1007/s11136-021-02788-3).

## Introduction

Heart failure (HF) is a pandemic syndrome affecting 1–2% of the adult population in developed countries [1]. Almost 6 million people in the US [2] and 15 million people in Europe [1] are affected by HF. As the prevalence increases with age, HF is projected to increase by 46% between 2012 and 2030 [1]. Patients with HF experience poor outcomes such as repeated hospitalization [1], high mortality rates [1, 2] and decreased quality of life [2, 3]. Quality of life (QOL) is compromised by the many symptoms caused by HF [3]. In addition to the well-known HF symptoms such as dyspnea, fatigue, poor exercise tolerance and fluid retention [4], there are other common symptoms of HF. In particular, anxiety and depression [5, 6] strongly affect sleep quality [7], thereby contributing to chronic insomnia [8, 9] and reduced QOL [10–13].

In addition to poor QOL, anxiety, depression and poor sleep quality are independently associated with increased hospitalization [14, 15] and mortality rates [15–17]. The prevalence and impact of these factors demonstrate the importance of identifying interventions that effectively reduce anxiety and depression and improve sleep quality and overall QOL. One intervention that has been shown to improve health behaviours and clinical outcomes is motivational interviewing (MI). Over 30 years of research have established MI, a patient-centred method for identifying and enhancing intrinsic motivation, as an effective technique for promoting behavioural change [18].

Originally developed as an effective approach for problem drinkers [19], MI has proven to be effective in a variety of patient populations (for example, smokers and patients with diabetes mellitus, HIV, cardiovascular disease and other conditions) [20–24]. The essence of MI is a supportive, eliciting, and empathic delivery style that can be used to empower patients to make healthy decisions [25]. In HF, MI has been used in combination with multidisciplinary behavioural intervention approaches such as psychological counselling, hospital discharge education, transitional care programs and telephone monitoring, with the aim of reducing hospital readmissions and improving health outcomes, through the promotion of relevant self-care behaviours [26].

Although two recent systematic reviews [25, 26] have evaluated the effectiveness of MI to improve QOL and psychological outcomes in HF patients, a lack of clarity about blinding, randomization, intervention fidelity, duration, dosage and training of interventionists has limited the ability to fully attribute outcomes to MI. Furthermore, the operant mechanism underlying intervention effectiveness remains unclear. That is, if MI improves QOL, the manner in which the intervention achieves this outcome is unclear. Although it is logical to consider improved sleep as one mechanism, only a few studies have used MI to modify sleep behaviours and improve sleep quality [27–29], and none of them were conducted on patients with HF. Interventions that effectively reduce anxiety and depression may improve sleep quality and QOL for people with HF. MI may also be suitable to directly address sleep-promoting behaviour. However, the effect of MI on anxiety, depression, sleep quality and QOL remains unclear.

To address these gaps in knowledge, the purpose of this study was to evaluate the effect of MI on anxiety, depression, sleep quality and QOL experienced by HF patients in the 12 months following receipt of an MI intervention.

## Methods

### Design

This was a planned analysis of a three-arm, randomized controlled trial (RCT) evaluating the improvements in patient self-care following MI in HF patients and caregivers (MOTIVATE-HF) [30]. The RCT, complied with the Declaration of Helsinki, was approved by the Institutional Review Board of the University of Rome “Tor Vergata”, and was registered at ClinicalTrials.gov (Identifier: NCT02894502) [30]. All participants provided written informed consent [31].

In this study, we evaluate the effect of the MI intervention on anxiety and depression, sleep quality, and both generic and disease-specific QOL. Study details have been described previously [30]. Aspects that are pertinent to this secondary analysis are summarized below.

### Participants

Adults with HF and their caregivers were recruited from three centres in the Lazio region of Italy: one hospital, one outpatient clinic, and one community setting. HF patients were assessed for study eligibility based on the following criteria. Those included in the study had the following characteristics: (1) a confirmed diagnosis of HF according to international guidelines [1]; (2) New York Heart Association (NYHA) functional class II–IV; (3) inadequate self-care when assessed against the Self-Care Heart Failure Index (SCHFI v6.2), with a score of 0, 1 or 2 in at least two items of the self-care maintenance or self-care management scales [32]; and (4) were willing to participate in the study. The exclusion criteria for patients were as follows: (1) severe cognitive impairment evaluated with a score of 0–4 on the six-item screener [33]; (2) an acute coronary syndrome event during the previous 3 months; (3) living in a residential setting (e.g. nursing home); or (4) caregiver was not willing to participate in the study.

Caregivers were included if they were willing to participate in the study, and if they were designated by the patient as the person in charge of informal primary care (that is, the person inside or outside the family caring for the HF patient). Caregivers were excluded if the patient was not willing to participate in the study. In cases when either the patient or the caregiver was not willing to participate in the study, both were excluded from enrolment. However, after enrolment, if one person dropped out, the other person continued in the study remaining in the same study arm. After enrolment, each patient–caregiver dyad was randomized to one of three study arms (1:1:1). In Arm 1, only patients received MI intervention, whereas in Arm 2, both patient and caregiver received MI intervention. Patients and caregivers in Arm 3 received standard care.

### Procedures, sampling, randomization and treatment fidelity

At the start of the study, socio-demographic, clinical (for example, NYHA functional class), comorbidity and cognition data were collected from all those enrolled. In addition, a battery of psychometrically sound instruments was administered independently to both patients and their caregivers. Follow-up assessment was performed by telephone at 3 (time 1, or T1), 6 (T2), 9 (T3) and 12 (T4) months after enrolment by nurse research assistants who were blinded to study arm assignment of participants, as were the investigators. Participants were not blinded to study arm.

The intervention was delivered by 18 registered nurses (different from the research assistants who collected the data), six in each centre, who attended a 40-h training course on HF care and MI. These 18 registered nurses were 13 female and 5 male nurses who were 38.3 years old on average (SD 10.4; range 25–59). The nurses had on average 5.7 years (SD 3.5; range 1–13) of clinical experience in cardiology. Eleven had a bachelor’s degree and seven had a nursing diploma. The intervention consisted of a face-to-face MI session (about 60 min in length) followed by 3 telephone conversations of about 15 minutes (within 2 months from enrolment). During the MI session, the interventionists applied the principles of MI [38] with the patient (Arm 1), or the patient and caregiver (Arm 2). In Arm 2, MI and telephone conversations for patients and caregivers were performed separately. The same standard of care (medical check-ups every 6–12 months depending on their HF condition, and information given orally on HF and its treatment) was also provided to patients and caregivers in Arms 1 and 2, as well as in Arm 3 (control group). The quality of all interventions was evaluated using audio recordings of each MI, published in detail elsewhere [30].

As reported in the primary outcome paper [30], a total of 510 HF patient and caregiver dyads were enrolled and randomized (1:1:1), with 155 patients and caregivers allocated to Arm 1, 177 allocated to Arm 2, and 178 allocated to Arm 3. Randomization was performed in the following way. First, a research assistant generated 1200 assignments (Arm 1, Arm 2 and Arm 3) following a block randomization scheme of 15 patient and caregiver dyads to guarantee balance among the three arms. Second, these 1200 assignments were put into opaque envelopes that were divided in three different containers (with 400 envelopes each). Then, the containers were distributed to the enrolling centres. At each centre, another research assistant opened an envelope when they received notification of an enrolment. That research assistant contacted the interventionist at that centre. The interventionist was instructed to perform MI only with the patient (Arm 1) or with the patient and caregiver (Arm 2).

The research assistants and the data collectors were blinded to patient and caregiver assignment to the study arms. The interventionists were not blinded to study arm assignment, but they did not collect data and could not influence the assignment of patients and caregivers to the study arms. Patients and caregivers were not blinded since they know the study arm to which they had been assigned. Participants who completed the full 12-month trial included 238 HF patients and 235 caregivers.

Treatment fidelity was evaluated with the Motivational Interviewing Treatment Integrity (MITI) Scale [39], which scores the technical and relational components of MI from 1 to 5, with higher scores indicating better MI quality. Ideally, technical and relational component scores should be ≥ 3 and ≥ 4, respectively. For this treatment fidelity analysis, the investigators randomly selected 48 interviews in Arm 1 and 97 interviews in Arm 2 (50 patient and 47 caregiver interviews) for audiotaping and rated the intervention after listening to the audiotaped sessions. The mean score of the technical component was 2.4 (SD, 0.5); the mean score of the relational component was 2.8 (SD, 0.8). Also, we checked whether the three telephone calls had been done after the first face-to-face intervention as planned. All the telephone calls had been done.

### Measurements

The tools and measures of the MOTIVATE-HF study [30] pertinent to the present analysis are described here. The Hospital Anxiety and Depression Scale (HADS) is commonly used to determine levels of anxiety and depression [40]. The HADS is a 14-item scale that generates 2 scores, with 7 items related to anxiety and 7 related to depression. Each item is scored from 0 to 3 with summary scores between 0 and 21 for anxiety and depression. A score of 8–10 indicates a moderate degree of symptoms, and a score ≥ 11 indicates a significant level of anxiety or depression.

The Pittsburgh Sleep Quality Index (PSQI) is a validated tool used to obtain self-reported sleep quality [41]. The PSQI contains 19 self-rated questions, and 5 additional questions rated by the bed partner or roommate, if available. Only the self-rated questions are included in the score [41]. The PSQI evaluates subjective sleep quality, sleep latency, sleep duration, habitual sleep efficiency, sleep disturbances, use of sleep medications and daytime dysfunction over the previous month. Each component generates a subscale score of 0–3, with 0 indicating no difficulty, and 3 indicating severe difficulty. These 7 scores are combined in one global score of 0–21 points (0 = no difficulty, 21 = severe difficulty) where a score of ≥ 5 indicates poor sleep quality [41]. In this analysis, we used the PSQI global score.

Generic physical and mental QOL was assessed using the 12-Item Short-Form Health Survey (SF-12) [42]. SF-12 is a multipurpose, short-form generic measure of health status, measuring physical functioning, role limitation (due to physical or emotional health problems), bodily pain, general health, vitality (energy and fatigue), social functioning and mental health (psychological distress and psychological well-being) [42]. Two summary scores are reported: a mental component score (MCS-12) and a physical component score (PCS-12). All scores are standardized in the range of 0–100, with higher scores indicating better mental and physical QOL [42].

The Kansas City Cardiomyopathy Questionnaire (KCCQ) [43] was used to measure HF-specific QOL. The KCCQ is a 23-item, self-administered questionnaire, developed to measure the patient’s perception of their health status. Questions address HF symptoms, impact on physical and social function, and how HF impacted their QOL in the previous 2 weeks. The KCCQ quantifies 6 distinct domains (symptoms, physical function, QOL, social limitation, self-efficacy and symptom stability), each with their own score, and two summary scores (clinical summary score and overall summary score) [43]. In this study, we used the overall summary score, the self-efficacy score and the symptom stability score. This choice was made because the overall summary score already includes four of the six domains scores: symptoms, physical function, social limitations and QOL. By using it in combination with the self-efficacy score and the symptom stability score, the evaluation is complete. The self-efficacy domain quantifies perceptions of how to prevent HF exacerbations and manage complications when they arise. The symptom stability domain measures recent changes in symptoms. Scores are summed within each domain. The overall summary, the self-efficacy and the symptom stability scores are all standardized in the range of 0–100 [43] with higher scores meaning better QOL.

For descriptive purposes, we considered the following clinical and socio-demographic characteristics of patients: gender; age; marital status; school education; employment; income; NYHA class [44]; comorbidities, as described by the Charlson Comorbidity Index (CCI) [45]; time since HF diagnosis; number of medications; cognition, as assessed by the Montreal Cognitive Assessment (MoCA) [46]; and self-care maintenance, management and confidence, as measured by the Self-care of Heart Failure Index version 6.2 (SCHFI V6.2) [32].

### Sample size

The target sample size of 480 patients, accounting for an estimated 50% attrition rate (80 per each arm at the end of follow-up), was based on self-care maintenance, the primary endpoint of the MOTIVATE-HF study [30]. Regarding the variables that we considered in this study, the above sample size was estimated to achieve 76% power to detect an effect size of 0.3 (minimally important difference of 1.5 [34] with a common standard deviation (SD) of 5) in HADS scores of patients receiving the MI intervention (Arms 1 and 2) vs. patients in usual care (Arm 3), with a significance level of 0.05 using a two-sided two-sample t-test. As far PSQI and SF-12, an effect size of 0.5 was expected resulting in 99% power (clinically significant change of 2 [35] with SD = 4 for PSQI and 5 [36] with SD = 10 for SF-12). The minimal clinically important difference in KCCQ scores is a 5-point improvement [37] with an expected SD of 18 resulting in power of 71%.

### Statistical analysis

Baseline characteristics were summarized by arm as median and interquartile range (IQR) or as mean and SD for continuous data, and as absolute number and percentage for categorical data. The change in HADS, PSQI, SF-12 and KCCQ scores during follow-up were reported as the difference (Δ) between each of these scores at each follow-up time (T1, T2, T3 and T4) and the corresponding scores at baseline (T0). A two-sample *t*-test was applied to compare the difference of each of these scores in Arms 1 and 2 with respect to the control Arm 3.

Changes over time (from T0 to T4) in the scores were analysed with mixed models to account for drop-out and missing values. As response variables, the HADS, Global PSQI, SF-12 and KCCQ scores available from T0 to T4 for each patient in the study arm were included. The dependence between HADS, Global PSQI, SF-12 and KCCQ scores on the same subject was accounted for by the inclusion of a random intercept and slope in each model. The models included as covariates the follow-up visit (as a continuous variable), the randomization arm (as a categorical variable), and the interaction between the randomization arm and the follow-up visit.

## Results

### Participant characteristics

Patient baseline characteristics, separated by study arm, are shown in Table 1. Patients overall were older adults with a higher proportion of men. Most were retired and minimally compromised in terms of functional class (NYHA Class II). The three groups were comparable at baseline in anxiety, depression, sleep quality and QOL. Specifically, moderate levels of anxiety and depression were identified, as well as poor sleep quality. Both generic and disease-specific QOL evaluation showed a moderate burden of symptoms (Table 1).

**Table 1:** Socio-demographic and clinical characteristics of HF patients at baseline (*n* = 510)

Characteristics	Missing	Arm 1: MI only for patients (*n* = 155)	Arm 2: MI for patients and caregivers (*n* = 177)	Arm 3: standard care for patients and caregivers (*n* = 178)
Gender (male), *n* (%)	0	80 (51.6)	107 (60.5)	109 (61.2)
Age, median (IQR)	2	74 (65–82)	73 (64–81)	75 (64–83)
Marital status, *n* (%)	0			
Married		81 (52.3)	123 (69.5)	112 (62.9)
Widowed		55 (35.5)	44 (24.9)	51 (28.7)
Divorced		10 (6.5)	4 (2.3)	6 (3.4)
Single		9 (5.8)	6 (3.4)	9 (5.1)
Education (high school or higher), *n* (%)	0	41 (26.4)	44 (24.8)	47 (26.4)
Employment (retired), *n* (%)	2	119 (76.8)	137 (77.8)	131 (74.0)
Income, *n* (%)	0			
Not the necessary to live		7 (4.5)	7 (4.0)	8 (4.5)
The necessary to live		131 (84.5)	138 (78.0)	141 (79.2)
More than the necessary to live		17 (11.0)	32 (18.1)	29 (16.3)
NYHA Class, *n* (%)	4			
II		98 (63.2)	108 (61.7)	107 (60.8)
III		49 (31.6)	55 (31.4)	56 (31.8)
IV		8 (5.2)	12 (6.9)	13 (7.4)
CCI Score, median (IQR)	0	2 (2–4)	2 (2–4)	2 (1–4)
Time with HF (months), median (IQR)	9	36 (24–72)	36 (15–84)	48 (20–96)
No. of medications, median (IQR)	9	6 (4–8)	7 (5–9)	6 (4–8)
MoCA Scores, median (IQR)	7	25 (21–27)	26 (19–28)	24 (18–27)
Self-Care Maintenance Scores, mean (SD)	0	45.72 (15.23)	45.98 (16.35)	44.98 (14.61)
Self-Care Management Scores^a^, mean (SD)	156	41.75 (17.94)	37.62 (18.43)	40.32 (16.40)
Self-Care Confidence Scores, mean (SD)	1	51.51 (20.94)	52.09 (21.24)	50.66 (22.56)
Hospital Anxiety Scale, median (IQR)	0	8 (4–11)	7 (5–11)	8 (5–11)
Hospital Depression Scale, median (IQR)	0	8 (4–10)	8 (5–10)	9 (5–12)
Global PSQI Score, median (IQR)	12	12 (10–15)	12 (9–15)	12 (9–14)
Physical SF-12 Health Survey, median (IQR)	0	33.8 (27.8–44.8)	33.7 (28.1–41.3)	34.3 (27.9–43)
Mental SF-12 Health Survey, median (IQR)	0	44.3 (36.9–53.1)	44 (39.2–52.3)	44.7 (37.9–52.6)
Kansas City Cardiomyopathy Questionnaire, median (IQR)				
Overall Summary Score^b^	0	44.8 (33.6–66.7)	50 (33.3–66.9)	48.2 (31.8–68)
Self-Efficacy Score	0	50 (37.5–62.5)	50 (25–75)	50 (37.5–75)
Symptom Stability Score	0	75 (50–100)	75 (50–100)	75 (50–100)

*MI* motivational interviewing, *IQR* interquartile range, *SD* standard deviation, *HF* heart failure, *CCI* Charlson Comorbidity Index, *MoCA* Montreal Cognitive Assessment, *NYHA* New York Heart Association, *PSQI* Pittsburgh Sleep Quality Index, *SF-12* Short Form (12), *KCCQ* Kansas City Cardiomyopathy Questionnaire

^a^Self-care management score can only be computed if patients have had HF symptoms in the last month (*n* = 354. Symptomatic patients were *n* = 100 in Arm 1, *n* = 130 in Arm 2, and *n* = 124 in Arm 3), and the percentages shown are the percentage of symptomatic participants in each arm

^b^Score includes the total symptom, physical function, social limitations and quality of life scores

**Table 2:** Changes in Hospital Anxiety and Depression Scale, Global PSQI, Physical and Mental SF-12 Health Survey, and KCCQ Overall Summary, Self-Efficacy and Symptom Stability Scores during follow-up, calculated by comparing to corresponding scores at T0

Variable	*N*	Arm 1: MI only for patients (*n* = 155)	Arm 2: MI for patients and caregivers (*n* = 177)	Arm 3: Standard of care (*n* = 178)	Difference (95% CI)^b^	*P*-value (Student’s *t*-test)
		Mean (SD)	Mean (SD)	Mean (SD)		
Δ in Hospital Anxiety Scale^a^						
T1	364	0.15 (4.95)	− 0.05 (3.91)	− 0.05 (4.12)	0.09 (− 0.86; 1.04)	0.8504
T2	292	− 0.76 (4.28)	− 1.64 (4.44)	− 1.41 (4.13)	0.17 (− 0.90; 1.24)	0.7580
T3	252	0.75 (4.24)	0.13 (4.37)	0.34 (4.43)	0.07 (− 1.09; 1.23)	0.9075
T4	238	− 0.45 (4.80)	− 1.73 (4.36)	− 0.43 (4.55)	− 0.72 (− 1.98; 0.53)	0.2594
Δ in Hospital Depression Scale^a^						
T1	364	0.07 (4.72)	0.27 (4.05)	− 0.53 (4.45)	0.71 (− 0.25; 1.67)	0.1485
T2	292	− 0.58 (3.54)	− 0.91 (3.82)	− 0.79 (4.24)	0.03 (− 0.93; ; 0.99)	0.9511
T3	252	0.92 (3.64)	0.82 (4.06)	0.44 (4.19)	0.42 (− 0.64; 1.48)	0.4318
T4	238	− 0.63 (3.75)	− 0.88 (4.23)	− 0.78 (4.44)	0.01 (− 1.13; 1.15)	0.9850
Δ in Global PSQI Score^a^						
T1	363	− 0.55 (2.99)	− 0.30 (3.27)	− 0.76 (3.54)	0.34 (− 0.38;1.06)	0.3566
T2	291	− 0.42 (2.90)	− 0.45 (3.30)	− 0.47 (3.66)	0.04 (− 0.78; 0.86)	0.9283
T3	249	− 0.10 (2.89)	− 0.94 (3.21)	− 0.35 (3.12)	− 0.22 (− 1.05; 0.62)	0.6115
T4	234	− 0.57 (2.65)	− 0.78 (3.48)	− 0.09 (3.25)	− 0.59 (− 1.47; 0.28)	0.1828
Δ in Physical SF-12 Health Survey^a^						
T1	364	0.68 (10.05)	2.15 (10.09)	1.12 (8.17)	0.35 (− 1.59; 2.29)	0.7238
T2	292	2.99 (8.02)	2.63 (9.29)	1.10 (7.96)	1.69 (− 0.42; 3.80)	0.1167
T3	252	1.38 (7.45)	2.89 (9.53)	0.04 (8.36)	2.17 (− 0.12; 4.46)	0.0634
T4	238	1.36 (8.43)	3.27 (10.72)	0.79 (8.67)	1.62 (− 0.97; 4.20)	0.2189
Δ in Mental SF-12 Health Survey^a^						
T1	364	1.33 (11.61)	1.24 (10.11)	1.57 (10.78)	− 0.29 (− 2.66; 2.08)	0.8116
T2	292	0.31 (11.96)	− 0.45 (11.72)	− 0.73 (10.64)	0.62 (− 2.23; 3.47)	0.6671
T3	252	3.00 (10.22)	4.44 (10.98)	2.29 (9.30)	1.49 (− 1.25; 4.23)	0.2840
T4	238	3.13 (10.46)	3.35 (11.69)	2.61 (10.54)	0.64 (− 2.36; 3.63)	0.6754
Δ in KCCQ Overall Summary Score^a^						
T1	364	1.3 (21.2)	0.7 (22.9)	0.8 (21.5)	0.12 (− 4.68; 4.92)	0.9613
T2	293	7.5 (17.7)	7.6 (21.0)	3.0 (16.5)	4.58 (− 0.06; 9.21)	0.0528
T3	252	11.0 (17.9)	13.2 (20.1)	5.5 (17.4)	6.73 (1.76; 11.71)	0.0082
T4	238	11.4 (18.6)	13.4 (22.3)	4.1 (17.9)	8.41 (2.98; 13.84)	0.0025
Δ in KCCQ Self-Efficacy Score^a^						
T1	364	1.5 (26.2)	1.9 (25.1)	− 0.3 (25.8)	2.05 (− 3.57; 7.68)	0.4733
T2	292	8.0 (24.0)	10.7 (24.1)	4.5 (19.3)	4.92 (− 0.28; 10.12)	0.0637
T3	252	10.9 (24.5)	14.0 (20.6)	4.9 (19.8)	7.67 (1.88; 13.46)	0.0096
T4	238	14.0 (25.1)	16.3 (24.7)	6.1 (20.7)	9.19 (2.73; 15.66)	0.0055
Δ in KCCQ Symptom Stability Score^a^						
T1	364	2.9 (32.0)	2.3 (38.3)	− 2.9 (34.8)	5.48 (− 2.25; 13.20)	0.1640
T2	293	− 4.1 (32.3)	6.3 (35.6)	− 4.1 (36.9)	5.73 (− 3.03; 14.49)	0.1990
T3	252	7.9 (30.4)	17.0 (33.4)	4.7 (29.4)	8.11 (− 0.29; 16.52)	0.0585
T4	238	7.9 (30.0)	14.6 (37.5)	5.3 (29.8)	6.31 (− 2.72; 15.35)	0.1701

*SD* standard deviation, *CI* confidence interval, *PSQI* Pittsburgh Sleep Quality Index, *SF-12* Short Form (12), *KCCQ* Kansas City Cardiomyopathy Questionnaire

^a^The columns for each arm report the delta (Δ) of each score, computed by subtracting the corresponding score at baseline from the corresponding score at each follow-up time (T1, T2, T3, T4)

^b^The difference is between Arm 1 and Arm 2 compared to Arm 3

**Fig. 1:**
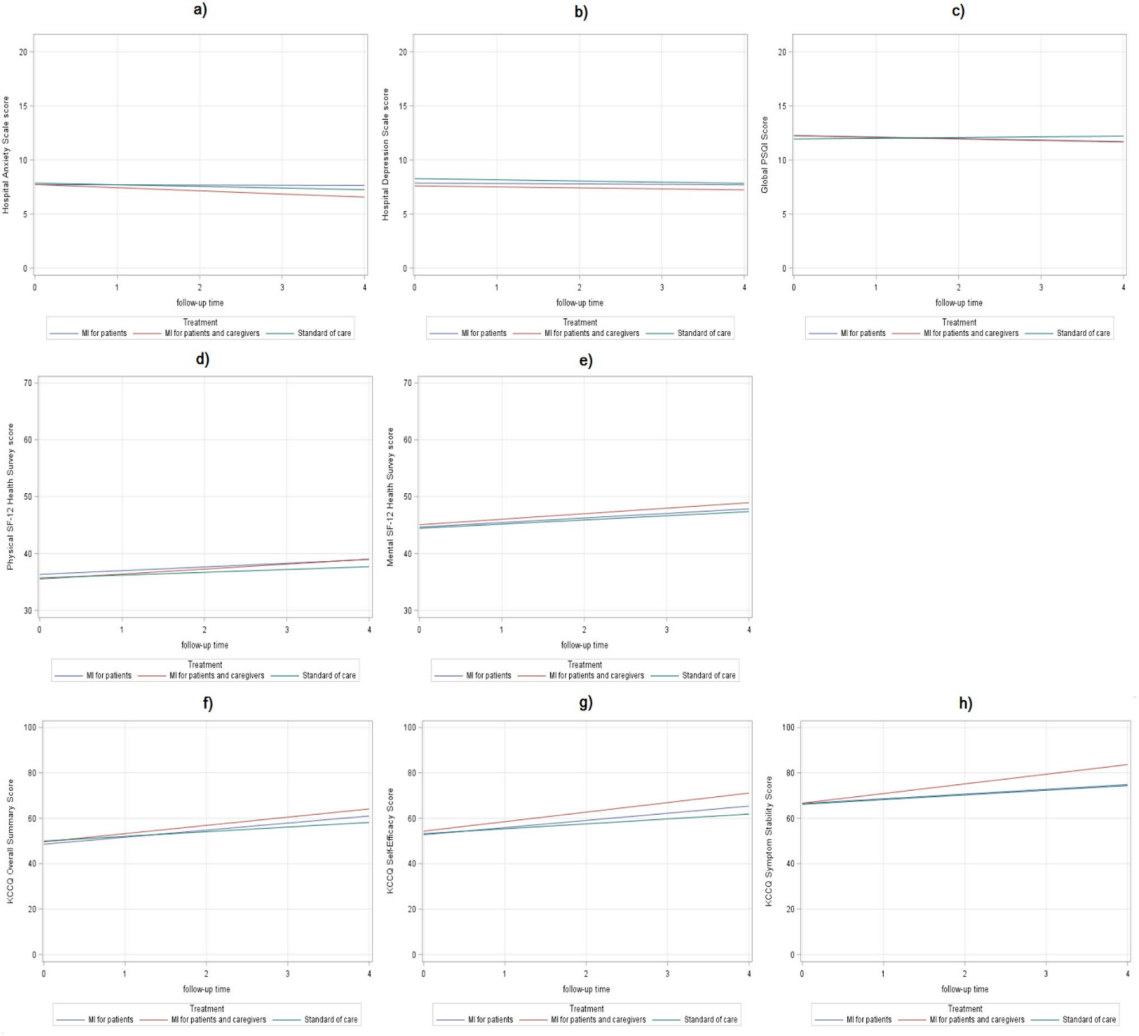
Model-based hospital anxiety (**a**) and Depression (**b**) Scale, Global PSQI (**c**), Physical (**d**) and Mental (**e**) SF-12 Health Survey, and KCCQ Overall Summary (**f**), Self-Efficacy (**g**) and Symptom Stability (**h**) Scores by follow-up time

### Anxiety and depression

No significant changes over time were seen in the anxiety or depression scores among the three study arms (Table 2). Model-based trends of HADS from baseline to T4 are shown in Fig. 1a and b. Over the year of the observation, neither anxiety nor depression improved more in either intervention arm (Arms 1 and 2) compared to Arm 3 (*P* = 0.35 and *P* = 0.32 in anxiety scale; *P* = 0.62 and *P* = 0.90 in depression scale). Moreover, there was no improvement of these scores over time (*β* = − 0.15, 95% CI (− 0.35; 0.05), *P* = 0.13 for anxiety and *β* = − 0.11, 95% CI (− 0.29; 0.07), *P* = 0.24 for depression).

### Sleep quality

No significant differences were observed in sleep quality across the three study arms over time (Table 2). In model-based trends, Arms 1 and 2 did not improve significantly more than Arm 3 (*P* = 0.09 and *P* = 0.07, respectively, Fig. 1c), with no improvement over time (*β* = 0.06, 95% CI (− 0.1; 0.23), *P* = 0.45).

### Quality of life

While there was an improvement in generic QOL (SF-12) scores over time, no significant differences were observed among the three arms (Table 2). Over the year of observation, neither Arms 1 nor 2 improved significantly more than Arm 3 (*P* = 0.63 and *P* = 0.23, respectively, for the SF-12 physical component summary, Fig. 1d; and *P* = 0.86 and *P* = 0.52, respectively, for the SF-12 mental component summary, Fig. 1e). However, there was differential improvement in the disease-specific QOL (KCCQ overall summary score), with significantly higher scores in the intervention Arms 1 and 2 compared to Arm 3 at T3 and T4 (difference 6.73, 95% CI (1.76; 11.71) and 8.41, 95% CI (2.98; 13.84), respectively) (Table 2). The longitudinal model (Fig. 1f), demonstrated an improvement over time in all three arms (*P* < 0.0001), with Arm 2 (patients and caregivers receiving MI intervention) having significantly greater improvement than Arm 3 (*β* = 1.57, 95% CI (0.26; 2.89), *P* = 0.02).

The improvement in KCCQ self-efficacy score was significantly greater in Arms 1 and 2 compared with Arm 3 at T3 and T4 (difference 7.67, 95% CI (1.88; 13.46) and 9.19, 95% CI (2.73; 15.66), respectively), but not at T1 and T2 (Table 2). When the three arms were analysed in the longitudinal model (Fig. 1g), Arm 2 showed more improvement than Arm 3 (*β* = 2.03, 95% CI (0.52; 3.55), *P* = 0.009).

No significant differences have been observed among the three arms regarding changes in KCCQ symptom stability score (Table 2). In the corresponding longitudinal model (Fig. 1h), once again, Arm 2 showed significantly more improvement with respect to Arm 3 (*β* = 2.19, 95% CI (0.09; 4.29), *P* = 0.04).

Longitudinal linear mixed model results on anxiety, depression, sleep quality and QOL are shown in Online Resource 1.

## Discussion

In this planned secondary analysis of data from the MOTIVATE-HF trial, we evaluated the effects of MI on anxiety, depression, sleep quality and QOL of patients with HF. We found that MI had no effect on anxiety, depression or sleep quality in the 12 months following the MI intervention. Only disease-specific QOL improved over time in the intervention arms compared to the control arm, but not until 9–12 months after the intervention. There may have been some differential improvement in the intervention arm that included both patients and caregivers, but this effect was not consistent across domains. These results are important because they emphasize three issues that deserve further study. Firstly, an MI intervention of this intensity may not be sufficient to improve mood disturbances. Secondly, the benefits of MI may be delayed in persons with HF. Thirdly, including the caregiver in the MI intervention may strengthen the effect.

The MI intervention significantly improved disease-specific QOL over time in the intervention groups later in the follow-up period. The domains with significant improvement were self-efficacy, symptom stability and the overall summary score. Our results are also clinically significant. Previous studies [37, 47] have estimated that the minimal clinically important difference in KCCQ scores is 5 points. In this study, in the KCCQ overall summary, self-efficacy and symptom stability scores, we observed a statistically significant improvement ranging between 6.72 and 9.19 points in the above three domains, even with a power of 71% in sample size. This means that our intervention had an important impact on disease-specific QOL. Self-efficacy improved in both intervention groups later in the follow-up period. This domain is probably better thought of as a knowledge or self-care domain rather than self-efficacy, as it refers to the belief that a patient has in their ability to perform and persist in performing a specific behaviours [48]. In fact, the KCCQ self-efficacy scale items address understanding of how to prevent symptoms, and what to do if HF worsens [43]. This understanding may have influenced the symptom stability scores, which also improved differentially in the intervention arm that included both patients and caregivers. With knowledge of how to prevent and manage symptoms, the MI intervention may have motivated shared self-care behaviours that improved symptoms over time. The overall summary score reflects physical limitations, symptoms, QOL and social limitations, and preferentially improved later in the follow-up period of the group where both the patient and caregiver received intervention. Again, this improvement appears to reflect support gained from the shared MI experience.

We were surprised that the MI intervention had no effect on anxiety or depression, as others have found that MI in hospitalized coronary heart disease patients was effective in decreasing depression two weeks later [49]. In another study in a sample of patients with HF, after four MI sessions and eight weeks of follow-up, depression was reduced [50]. It may be that our MI intervention was not intense enough to affect a variable like mood.

The patients in this study had poor sleep quality on enrolment, and the MI intervention had no effect on sleep quality over time. This finding is similar to that found in a study on motivational intervention for adolescent sleep problems. Those investigators provided four, weekly, 50-min sleep education classes and found that subjects had an increased knowledge about sleep, but showed no improvements in sleep quality or daytime functioning [28]. Sleep may not be directly amenable to an intervention designed to change volitional behaviour. That is, approaches that emphasize something other than motivation to sleep better are probably needed to improve the quality of sleep, particularly in those with a chronic condition such as HF.

### Limitations and strengths

Limitations of this study include issues with intervention fidelity. As discussed in the primary study report, technical and relational issues were identified with MI quality [30]. When treatment integrity was evaluated, both the technical and relational components of the intervention were judged to be somewhat lower than desired, which may have compromised our ability to see an effect of the intervention on these secondary outcomes. However, this is one of the few studies assessing this effect, and we used a number of valid and reliable tools to measure the outcomes of interest. The trial was also multi-centred which supports the rigour of the obtained results.

## Conclusion

In this study, MI had no effect on anxiety, depression or sleep quality experienced by HF patients in the 12 months following the intervention. Future research will need to evaluate the intensity of MI interventions for them to be effective on these factors. However, MI was effective in improving disease-specific QOL, especially in the arm where both patient and caregiver received the intervention. Thus, clinicians might want to include both patients and caregivers when providing MI interventions. Considering the association between anxiety, depression and poor sleep quality with hospitalization and mortality rates in people with HF, future research will need to develop effective interventions to improve these factors.

## Supplementary Information

Below is the link to the electronic supplementary material.

Supplementary file1 (DOCX 22 KB)
