# Supplementary material for: Effectiveness of motivational interviewing on anxiety, depression, sleep quality and quality of life in heart failure patients: secondary analysis of the MOTIVATE-HF randomized controlled trial
Source: Qual Life Res. 2021 Feb 22;30(7):1939–49. doi: 10.1007/s11136-021-02788-3 (PMC8233269; doi:10.1007/s11136-021-02788-3)
Supplement: Supplementary file 1 — Supplementary file1 (DOCX 22 KB) [file 11136_2021_2788_MOESM1_ESM.docx]

**Supplementary Information (SI)**

**Online Resource 1** Longitudinal linear mixed model results on Hospital Anxiety and Depression Scale, Global Pittsburgh Sleep Quality Index Score, Physical and Mental SF-12 Health Survey, and Kansas City Cardiomyopathy Questionnaire-Overall Summary, Self-Efficacy and Symptom Stability scores

| **Effect** | **β** | **95% CI** | **P-value** |
| --- | --- | --- | --- |
| **Hospital Anxiety Scale** |  |  |  |
| Time (for each visit) | -0.1538 | (-0.3539; 0.0464) | 0.1317 |
| MI only for patients vs standard care | -0.1412 | (-0.9394; 0.6570) | 0.7285 |
| MI for patients and caregivers vs standard care | -0.1346 | (-0.9057; 0.6365) | 0.7319 |
| Time * MI only for patients | 0.1347 | (-0.1507; 0.4202) | 0.3543 |
| Time * MI for patients and caregivers | -0.1382 | (-0.4113; 0.1350) | 0.3210 |
| **Hospital Depression Scale** |  |  |  |
| Time (for each visit) | -0.1083 | (-0.2905; 0.0739) | 0.2433 |
| MI only for patients vs standard care | -0.4043 | (-1.1989; 0.3902) | 0.3181 |
| MI for patients and caregivers vs standard care | -0.6668 | (-1.4344; 0.1008) | 0.0885 |
| Time * MI only for patients | 0.0663 | (-0.1935; 0.3260) | 0.6167 |
| Time * MI for patients and caregivers | 0.0156 | (-0.2330; 0.2642) | 0.9017 |
| **Global PSQI Score** |  |  |  |
| Time (for each visit) | 0.0644 | (-0.1016; 0.2304) | 0.4462 |
| MI only for patients vs standard care | 0.3139 | (-0.4383; 1.0662) | 0.4129 |
| MI for patients and caregivers vs standard care | 0.2854 | (-0.4399; 1.0106) | 0.4401 |
| Time * MI only for patients | -0.2035 | (-0.4396; 0.0326) | 0.0911 |
| Time * MI for patients and caregivers | -0.2055 | (-0.4307; 0.0198) | 0.0737 |
| **Physical SF-12 Health Survey** |  |  |  |
| Time (for each visit) | 0.4949 | (0.0378; 0.9519) | 0.0339 |
| MI only for patients vs standard care | 0.6284 | (-1.1988; 2.4556) | 0.4998 |
| MI for patients and caregivers vs standard care | -0.1957 | (-1.9608; 1.5694) | 0.8278 |
| Time * MI only for patients | 0.1584 | (-0.4912; 0.8080) | 0.6323 |
| Time * MI for patients and caregivers | 0.3831 | (-0.2383; 1.0044) | 0.2265 |
| **Mental SF-12 Health Survey** |  |  |  |
| Time (for each visit) | 0.7346 | (0.1981; 1.2710) | 0.0074 |
| MI only for patients vs standard care | 0.2091 | (-1.7149; 2.1331) | 0.8311 |
| MI for patients and caregivers vs standard care | 0.6252 | (-1.2333; 2.4837) | 0.5092 |
| Time * MI only for patients | 0.0683 | (-0.6954; 0.8320) | 0.8607 |
| Time * MI for patients and caregivers | 0.2367 | (-0.4939; 0.9672) | 0.5250 |
| **KCCQ Overall Summary Score** |  |  |  |
| Time (for each visit) | 2.0501 | (1.0849; 3.0153) | <0.0001 |
| MI only for patients vs standard care | -1.4082 | (-5.7675; 2.9511) | 0.5262 |
| MI for patients and caregivers vs standard care | -0.3535 | (-4.5647; 3.8578) | 0.8692 |
| Time * MI only for patients | 1.0573 | (-0.3157; 2.4302) | 0.1310 |
| Time * MI for patients and caregivers | 1.5728 | (0.2597; 2.8859) | 0.0190 |
| **KCCQ Self-Efficacy Score** |  |  |  |
| Time (for each visit) | 2.1577 | (1.0447; 3.2706) | 0.0002 |
| MI only for patients vs standard care | -0.4741 | (-4.7063; 3.7581) | 0.8260 |
| MI for patients and caregivers vs standard care | 1.1087 | (-2.9796; 5.1971) | 0.5946 |
| Time * MI only for patients | 0.9975 | (-0.5883; 2.5833) | 0.2173 |
| Time * MI for patients and caregivers | 2.0344 | (0.5169; 3.5518) | 0.0087 |
| **KCCQ Symptom Stability Score** |  |  |  |
| Time (for each visit) | 2.0595 | (0.5185; 3.6005) | 0.0089 |
| MI only for patients vs standard care | 0.3393 | (-5.9465; 6.6252) | 0.9156 |
| MI for patients and caregivers vs standard care | 0.4971 | (-5.5749; 6.5690) | 0.8724 |
| Time * MI only for patients | 0.0243 | (-2.1722; 2.2207) | 0.9827 |
| Time * MI for patients and caregivers | 2.1880 | (0.0868; 4.2891) | 0.0413 |

MI = Motivational Interview; PSQI=Pittsburgh Sleep Quality Index; KCCQ=Kansas City Cardiomyopathy Questionnaire
